# Characterization of S3Pvac Anti-Cysticercosis Vaccine Components: Implications for the Development of an Anti-Cestodiasis Vaccine

**DOI:** 10.1371/journal.pone.0011287

**Published:** 2010-06-23

**Authors:** Dunia Rassy, Raúl J. Bobes, Gabriela Rosas, Victor H. Anaya, Klaus Brehm, Beatriz Hernández, Jacquelynne Cervantes, Saúl Pedraza, Julio Morales, Nelly Villalobos, Aline S. de Aluja, Juan P. Laclette, Caris M. Nunes, Germano F. Biondi, Gladis Fragoso, Marisela Hernández, Edda Sciutto

**Affiliations:** 1 Departamento de Inmunología, Instituto de Investigaciones Biomédicas, Universidad Nacional Autónoma de México, Distrito Federal, México; 2 Facultad de Medicina, Universidad Autónoma del Estado de Morelos. Cuernavaca, Morelos, México; 3 Institute for Theoretical Biology, Humboldt Universität zu Berlin, Berlin, Germany; 4 Institut für Hygiene und Mikrobiologie, Julius-Maximillians-Universität Würzburg, Würzburg, Germany; 5 Facultad de Medicina, Universidad Nacional Autónoma de México, Distrito Federal, México; 6 Facultad de Medicina Veterinaria y Zootecnia, Universidad Nacional Autónoma de México, Distrito Federal, México; 7 Departamento de Apoio, Produção e Saúde Animal, Campus de Araçatuba, Universidad Estadual Paulista “Júlio de Mesquita Filho, Araçatuba, São Paolo, Brazil; 8 Departamento de Higiene Veterinária e Saúde Pública, Faculdade de Medicina Veterinária e Zootecnia de Botucatu, Campus Botucatu, Universidad Estadual Paulista “Júlio de Mesquita Filho, Botucatu, São Paolo, Brazil; The George Washington University Medical Center, United States of America

## Abstract

**Background:**

Cysticercosis and hydatidosis seriously affect human health and are responsible for considerable economic loss in animal husbandry in non-developed and developed countries. S3Pvac and EG95 are the only field trial-tested vaccine candidates against cysticercosis and hydatidosis, respectively. S3Pvac is composed of three peptides (KETc1, GK1 and KETc12), originally identified in a *Taenia crassiceps* cDNA library. S3Pvac synthetically and recombinantly expressed is effective against experimentally and naturally acquired cysticercosis.

**Methodology/Principal Findings:**

In this study, the homologous sequences of two of the S3Pvac peptides, GK1 and KETc1, were identified and further characterized in *Taenia crassiceps* WFU, *Taenia solium*, *Taenia saginata*, *Echinococcus granulosus* and *Echinococcus multilocularis*. Comparisons of the nucleotide and amino acid sequences coding for KETc1 and GK1 revealed significant homologies in these species. The predicted secondary structure of GK1 is almost identical between the species, while some differences were observed in the C terminal region of KETc1 according to 3D modeling. A KETc1 variant with a deletion of three C-terminal amino acids protected to the same extent against experimental murine cysticercosis as the entire peptide. On the contrary, immunization with the truncated GK1 failed to induce protection. Immunolocalization studies revealed the non stage-specificity of the two S3Pvac epitopes and their persistence in the larval tegument of all species and in *Taenia* adult tapeworms.

**Conclusions/Significance:**

These results indicate that GK1 and KETc1 may be considered candidates to be included in the formulation of a multivalent and multistage vaccine against these cestodiases because of their enhancing effects on other available vaccine candidates.

## Introduction

Parasitic zoonoses such as cysticercosis and hydatidosis seriously affect human health and are responsible for considerable economic loss in animal husbandry in developing and developed countries [1]. Cestode lifecycles were established in their respective hosts during the Pleistocene or earlier [2], and such long coexistence has evolved into an intricate, multifaceted relationship. To interrupt transmission of disease-causing cestodes to humans, simultaneous and sustained implementation of far-reaching measures and comprehensive social programs are required. These include devising and implementing specific and general health education programs, improving living conditions, and treating tapeworm carriers, among others. Vaccination represents an additional tool for control and prevention. Various whole or subunit vaccine candidates have been tested with promising protective effects against cysticercosis due to *T. solium* and *T. saginata*, and against hydatid disease caused by *E. granulosus* and *E. multilocularis* [3]–[10]. Their effectiveness, however, has been assessed mostly in controlled experimental environments rather than in complex natural conditions. Additionally, renewed hope on the successful formulation of vaccines for the prevention of intestinal infections by adult *Taenia* species has recently emerged [11]–[14].

Our research group developed the anti-cysticercosis S3Pvac vaccine [15]–[18]. S3Pvac is composed of three peptides, namely KETc12, KETc1, and GK1 of 8, 12, and 18 amino acids, respectively. Their protective capacity was first observed in a *T. crassiceps* murine cysticercosis model [16], [17]. The peptides were derived from native antigens present in different developmental stages of *T. crassiceps*. Using specific antibodies it was found that these peptides are also present in various *T. solium* anatomical structures [16], [17]. S3Pvac also induces significant protection against cysticercosis in vaccinated pigs, both experimentally and in the field where transmission conditions are most pressing [18]. It also induces high levels of protection against experimental tapeworm infection in hamsters [12].

A second S3Pvac version, S3Pvac-phage, has been developed by expressing the peptides in filamentous phages [19]. S3Pvac-phage reduced the frequency of tongue cysticercosis by 70%. A decrease of 54% and 87% in pig infection frequency and in the number of cysticerci, respectively, was detected in necropsies of 1,047 rural pigs from 16 villages of central Mexico [20]. S3Pvac-phage is now being produced massively and included in ongoing Mexican regional control programs.

The extensive similarities among cestodes regarding their natural history, pathology, and antigenic composition [21]–[23] often allow us to extend the use of a protective antigen against one species to others [5], [24]. Besides, many antigens are shared among the different developmental stages of cestodes (i.e. eggs, cysticerci, tapeworms) [16], [17], [25], [26]. These inter-species and inter-stage shared antigens offer an interesting common target for vaccine design. In this study, the homologous sequences of two of the S3Pvac peptides were identified in different cestodes, including *E. granulosus*, *E. multilocularis*, and *T. saginata*, with the aim of developing a single vaccine against cysticercosis and hydatidosis.

## Results

### S3Pvac presence in the four cestodes

**Figure 1:**
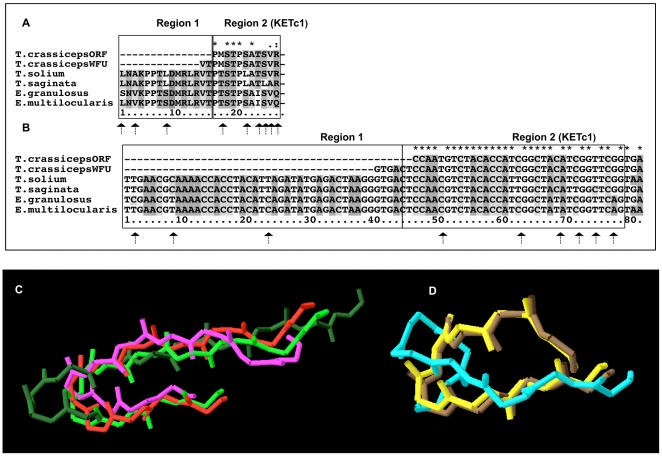
Multiple sequence alignment of KETc1 peptides. The DNA and amino acid sequences of KETc1 of several species of the Taeniidae family were aligned. KETc1 alignments of amino acids (A) and cDNA (B) are shown in the upper part of the figure. On every alignment, boxes corresponding to the regions subjected to dN/dS-ratio analysis are marked accordingly. Panel C and D shows the 3D models of the complete and truncated version of KETc1. Panel D depicts the models corresponding to *T. solium* (red), *T. crassiceps* ORF and WFU (pink), *T. saginata* (light green) and *Echinococcus* group (dark green). Structures are aligned with very good general agreement. Panel D shows the alignment of the truncated KETc1 peptide where the yellow model corresponds to *T. solium* and *T. saginata*; blue corresponds to *T. crassiceps* ORF and WFU and brown to the *Echinococcus* group.

Figure 1 shows multiple alignments of deduced amino acid sequences (A) and encoding DNA (B) for KETc1. For the sake of simplicity, KETc1 alignment was divided into two regions: the first includes the alignment before the KETc1 sequences, and the second includes the vaccine peptide. In Region 1, three non-synonymous mutations were found, as marked with arrows. Region 2 (KETc1) shows six amino acid changes among the different species and strains.

The dN/dS test of regions 1 and 2 (see Materials and Methods) revealed signs of positive selection regions, which could indicate that divergent evolution is taking place at these loci.

**Figure 2:**
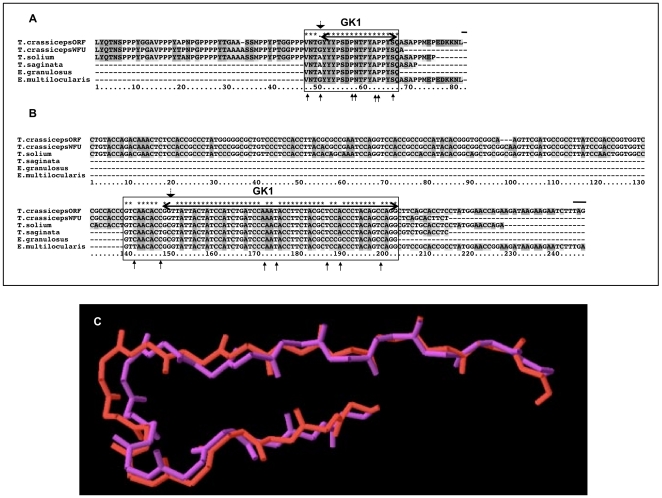
Multiple sequence alignment GK1 peptides. The DNA and amino acid sequences of GK1 in several species of the Taeniidae family were aligned. GK1 alignments of amino acids (A) and cDNA (B) are shown in the upper part of the figure; the region encoding the actual peptides is marked with a double-headed arrow on the top of the alignment. On every alignment, boxes corresponding to the regions subjected to dN/dS-ratio analysis are marked accordingly. Models of the 3D structure of the GK1 peptide are shown in panel C. The model in red corresponds to *T. solium*, *T. saginata* and *E. granulosus*' GK1 and in purple to *T. crassiceps* ORF and WFU and *E. multilocularis*. A very good fitting between the two structures is shown as expected from the strong similarity of the amino acid sequence.

Figure 2 shows the multiple alignments of the highly conserved sequence of GK1. One amino acid change was detected (big-headed arrow), and seven synonymous mutations were found (little-headed arrows). At the end of the alignment (Panels A and B) a black line marks the end of the protein including the peptide. The dN/dS analysis reveals signs of purifying selection acting on these regions (dN/dS <1).

### 3D structure prediction of KETc1 and GK1

Four models for KETc1 were constructed using the sequences listed in Table 1. Panel C in Figure 1 shows the alignment of 3D models corresponding to the whole peptide sequence for *T. solium* (red), *T. crassiceps* ORF and WFU (pink), *T. saginata* (light green), and the *Echinococcus* group (dark green). The five sequences are evidently similar in their general structure, except those corresponding to the *Echinococcus* group. These differences were expected considering the differences at sequence level among species. The models corresponding to the truncated version of KETc1 are shown in panel D, while the model in yellow corresponds to *T. solium* and *T. saginata*; brown corresponds to the *Echinococcus* group and blue to *T. crassiceps* ORF and WFU. Here, the sequence differences between *T. solium* and *T. saginata* disappeared. Differences in sequence (and structure) still exist in the case of *T. crassiceps* ORF and WFU and the *Echinococcus* group, although for the latter, they are not reflected in the modeled 3D structure (panel D).

**Table 1:** Sequences used to model the 3D structure of KETc1, GK1 and KETc12.

Peptide	Organism	Sequence
GK1	*T. solium*/*T. saginata*/*E. granulosus*	vntAYYYPSDPNTFYAPPYSQ
	*T. crassiceps* ORF/*T. crassiceps* WFU/*E. multilocularis*	vntGYYYPSDPNTFYAPPYSQ
KETc1^1^	*T. crassiceps* 2 ORF/WFU	kpptldmrlrvtPMSTPSATSVR
	*T. solium*	tldmrlrvTPTSTPLATSVRkpp
	*T. saginata*	kpptldmrlrvTPTSTPLATLAR
	*E. multilocularis*/*E. granulosus*	kpptsdmrlrvTPTSTPSAISVQ
KETc1- truncated	*T. crassiceps* ORF/WFU	kpptldmrlrvTPMSTPSAT
	*T. solium*/*T. saginata*/	kpptldmrlrvTPTSTPLAT
	*E. multilocularis*/*E. granulosus*	kpptsdmrlrvTPTSTPSAI

1 GK1 and KETc1 3D models include more than just the peptide sequence because the HMMSTR-server requires sequences of at least 20 amino acids length. Upper case marks the sequences of peptides while lower caps mark the preceding sequence to them.

2 There is no “complete” sequence for any of the strains of *T. crassiceps.* However, considering the strong similarity between the *Taenia* species we decided to construct a hypothetical sequence for each strain assuming that the preceding regions to KETc1 in *T. crassiceps* spp. are identical to those preceding the peptide in the other *Taenia* species.

The high conservation level in the amino acid sequence of GK1 is well reflected in the models shown in Panel C of Figure 2. The two variants in the sequence of GK1, group the organisms in *T. solium*, *T. saginata*, and *E. granulosus* and *T. crassiceps* ORF and WFU, and *E. multilocularis* are shown. The model shows numerous similarities in the structure of the GK1 peptide from different cestodes.

### S3Pvac expression in the four cestodes

**Figure 3:**
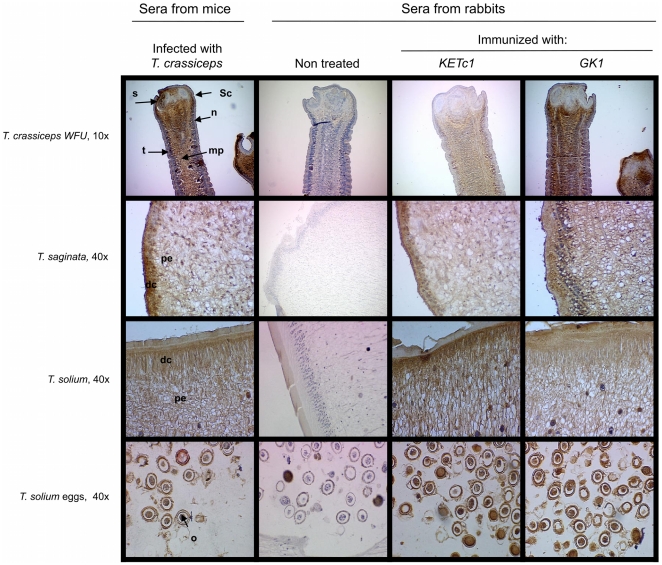
Immunolocalization of the S3Pvac peptides in larval specimens of the four Taeniidae families. Metacestode cryostat parasite sections were incubated with a rabbit pre-immune serum (A) or with the three different rabbit antibodies (anti-GK1, anti-KETc1, anti-KETc12) and developed with biotinylated goat anti-rabbit IgG plus streptavidin peroxidase conjugate and counterstained with hematoxilin. The arrows indicate the areas in the metacestode where the peptides were localized. 10X (A and C), 40X (B and D). Tegument (**T**), parenchyma (**P**), tegument of the spiral canal (**TSC**), parenchymal folds (**PF**), microthrix (**MT**), parenchyma (**P**). The three peptides are present in metacestode stage of four cestodes analyzed.

**Figure 4 pone-0011287-g004:**
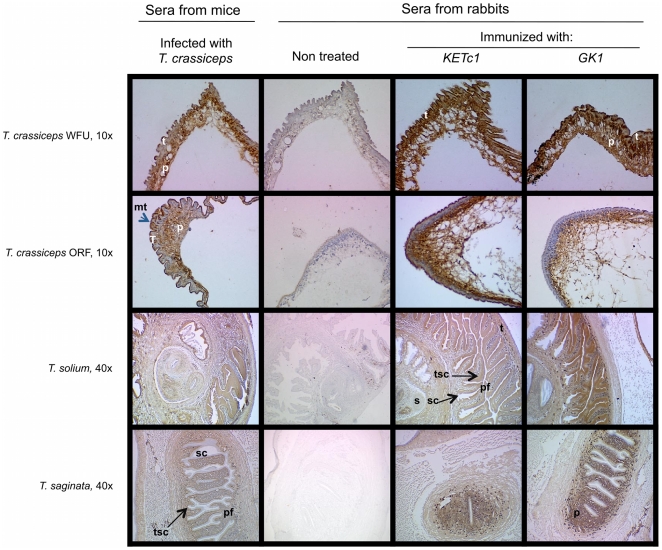
S3Pvac peptides were detected in the tegument of *Taenia* tapeworms and *T. solium* eggs. No reaction was detected in control sections, for which naive rabbit serum was used in place of the specific polyclonal rabbit serum. **S.**- Sucker; **Sc.** Scolex, **MP.**- Medullar parenchyma, **N.**- Neck; **DC.**- Distal cytoplasm region; **PE.**- perinuclear cytoplasm region. **O.**- oncospheres.

**Figure 5:**
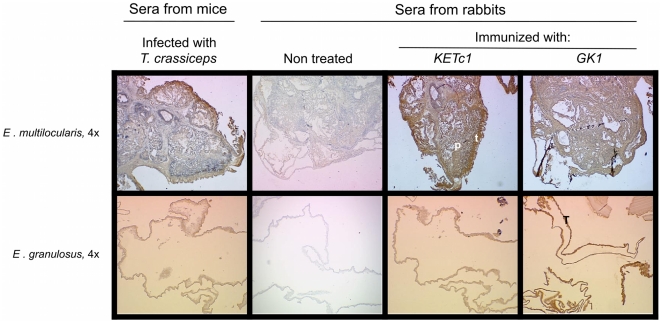
Immunolocalization of the S3Pvac peptides in *E. granulosus* and *E. multilocularis*. Sections of larval specimens reveal a strong binding to the tegument (**T**) as well as in the parenchyma (**P**) of *E. multilocularis* and in tegument of *E. granulosus*. (**A**) Control labeled with rabbit pre immune serum. (**B**) Positive control labeled with polyclonal sera from rabbit immunized with a total extract of *Taenia crassiceps* ORF cysticerci.

Figures 3, 4 and 5 show the expression of KETc1 and GK1 in the different cestodes and throughout the developmental stages of the parasites. Figure 3 shows the immunolocalization of the native protein in the adult stage of *T. crassiceps* WFU, *T. solium*, and *T. saginata*, and in *T. solium* oncospheres. GK1 was extensively expressed in the tissues of the three adult stages of the species analyzed. It was also localized in the parenchyma and tegument of *T. crassiceps*; and in the distal cytoplasm (DC) region and the perinuclear cytoplasm (PC) of *T. saginata* and *T. solium* tapeworms. GK1 was also expressed in the oncosphere (O) of *T. solium* eggs. KETc1 was only lightly detected in the medullar parenchyma (MP) of adult tissues of *T. crassiceps*. In *T. saginata* and *T. solium*, KETc1 was detected mainly in DC, PE, and in the oncosphere. Immunolocalization of the peptides in the larval phase of the parasites is shown in Figure 4. The two peptides were present in the cysticerci of both strains of *T. crassiceps*, but with different localization. In the cysticerci of WFU strain, the three peptides were located in MT, T, and P, while in those of ORF strain only KETc1 was located in the tegument, specifically in the MT of the tegument. In contrast, the two peptides were located in the parenchyma. In *T. solium* cysticerci, GK1 presence in the tegument of the spiral canal (TSC) and in tegument (T) seemed to be strong; a similar distribution was observed when antibodies against GK1 were employed. In metacestodes of *T. saginata*, KETc1 was detected in the parenchymal folds (PF) and lightly in TSC. GK1 was present in the parenchyma, but not in the TSC.

Both peptides were detected in the tegument of *E. granulosus* and *E. multilocularis*, but with some differences (Figure 5). KETc1 was more intensely detected than GK1 in *E. multilocularis*, whilst GK1 was the most conspicuous in *E. granulosus*.

### Effect of truncation in the protective capacity of KETc1 and GK1 peptides

Differences in the three terminal amino acids were found between *Taenia* and *Echinococcus* specimens of KETc1 and between *T. saginata* and the other taenids. To evaluate the biological relevance of these differences, a truncated version of KETc1, in which the last three amino acids were deleted, was employed. As Table 2 shows, the 9 amino acid-long new peptide maintained its protective capacity when compared with the full-length peptide. KETc1 and its truncated version significantly reduced the expected number of cysticerci in 68.7 and 69.3%, respectively.

**Table 2:** Protection against experimental murine cysticercosis induced by *T. crassiceps* derived KETc1 or GK1 peptides or their truncated analogs.

	Individual number of cysticerci per mouse	Mean ± SD
Naïve	186, 62, 189, 92, 5, 55, 101, 144, 149	109±62.7 ^a^
(non-immunized controls)		
Mice immunized with:		
KETc1 (APMSTPSATSVR)	0, 28, 14, 124, 0, 16, 72, 19	34.1±42.8 ^b^
KETc1 truncated (APMSTPSAT)	73, 0, 17, 0, 0, 6, 126, 46	33.5±45.8 ^b^
GK1(GYYYPSDPNTFYAPPYSA)	25, 22, 37, 63, 60, 1, 12	31.4±23.4 ^b^
GK1 truncated(GYYYPSDPNT)	1, 2, 95, 77, 2, 0, 135, 193	63.1±74 ^a^

Cysticerci were recovered 30 days after challenge. Different superscript letters indicate the statistically significant differences (*P*<0.05) by a Student's *t*-test.

GK1 was almost identical in the different specimens tested. However, considering that it is an 18-aa peptide, we decided to evaluate the relevance of the terminal amino acid composition by using a peptide including only the 10 first amino acids. Mice immunized with GK1 reduced the expected number of cysticerci by 71%, whilst the truncated GK1 did not reduce the number of cysticerci compared to naïve control mice (Table 2).

All recovered cysticerci were vesicular and motile under the stereoscopic microscope.

## Discussion

The S3Pvac anti-cysticercosis vaccine, composed of three peptides of 8 (KETc12), 12 (KETc1), and 18 amino acids (GK1) [16], [17], [27], was first identified in a *T. crassiceps* strain ORF cDNA library [28]. In this study we provide evidence indicating that two of these peptides, KETc1 and GK1, are highly conserved among different cestodes belonging to the genera *Taenia* (*T. solium* and *T. saginata*) and *Echinococcus* (*E. granulosus* and *E. multilocularis*). These results are not unexpected since extensive similarities among cestodes [21]–[23] have been largely demonstrated in the past, and offer the possibility of using the respective antigen homologues for protecting against other cestodiases [5], [24], [29]. These similarities observed among cestodes are encouraging, in particular considering that synthetically produced S3Pvac decreased the total number of *T. solium* cysticerci (98.7%) and reduced the prevalence (52.6%) of pig cysticercosis in different field trials performed in rural communities of Mexico [15], [18]. More recently, it has also been proved that S3Pvac recombinantly expressed in M13 filamentous phages also reduced pig cysticercosis (54%) and the number of established cysticerci by 87% [20]. Thus, these results allow us to propose the possible efficacy of these peptides if they are included in a multi-epitope vaccine against the aforementioned cestodes that, as mentioned before, cause serious human ailments and substantial economic loss to animal husbandry in developing and developed countries. In order to improve vaccine efficiency against cestodes, a vaccine formulation based on multistage epitopes, such as GK1 and KETc1, should be considered. This strategy has been successfully used against other parasitic diseases [30]. Considering this purpose, it would also be of interest to include the oncosphere TOVIS18 vaccine candidate. This oncospheral antigen and its homologues in other taenids (TSOL18 and TSAG18/HP6 from *T. solium* and *T. saginata*, respectively) exhibit substantial conservation of their gene structure. It has recently been reported that TSOL18 induced an amazing 100% of protection against the homologous infection [31]. Lower but still significant protection of 74% was also induced when TOVIS18 was used in combination with two additional recombinant vaccines against pig cysticercosis [5], [29], [31]–[34]. Taken together, these results encourage us to propose the use of KETc1 and GK1, and the respective HP6 homologues, in the design of polyvalent vaccines against cestodes, considering their protective capacity, and their presence in different cestode species and in different parasite stages and structures. In addition, it would be possible to kill oncospheres as well as early cysticerci, which may evade the initial immune attack.

Looking closely at the homologous sequences described herein, we found that most of the differences lie in the two strains of *T. crassiceps*. Particularly, the ORF strain has unique point mutations and major changes in the KETc1 reading frame. The latter causes the introduction of non-sense codons in what appears to be an open reading frame. Other point mutations are also found in GK1. GK1 is a very well conserved peptide among the studied species, but the surrounding region shows differences between the ORF and WFU strains. However, based on the analysis of available sequences, it is clear that, as in the case of KETc1, *T. crassiceps* ORF may be the most divergent strain. This is not completely unexpected, since the ORF strain was originally obtained in 1962 [35] and has been reproduced by budding in the peritoneal cavity of mice since then. This asexual multiplication has resulted in the loss of the capacity for scolex production and of two chromosomes [36]. These aberrations lead to the production of sterile cysticerci, incapable of developing into adult tapeworms in the definitive host [35]. This strain has been maintained at our Institute and specifically for our research group under passage of cysticerci in the peritoneal cavity of female BALB/cAnN mice every two months since 1986 [37]. In contrast, the WFU strain was recently isolated, and although it has also been maintained by intraperitoneal passage, it has retained its capacity to develop into the adult stage [38].

3D models of the peptides presented in this report were produced using an ab-initio predicting tool. This means that every time a sequence is modeled there are no assumptions on homology, and the model is built “from scratch”, based only on information from physicochemical forces. Differences between molecules may arise not only as a result of differences in sequence, but also as a consequence of the modeling process itself. Nevertheless, we believe that these models are a good information source, relevant for vaccine design and functionality prediction. The 3D models of KETc1 show some of the effects that changes in the amino acid sequences could produce. Among the *Taenia* group sequences, that of *T. crassiceps* ORF presents the most divergent structure. Such differences persist even when a truncated version of the peptide is modeled (Figure 1, Panel D), a circumstance under which all the other peptides seem to adopt very similar configurations.

Murine *T. crassiceps* cysticercosis was used as a model to test the biological significance of the differences in the KETc1 amino acid content. This model has been extensively used to identify promising antigens for vaccines against cysticercosis [24], [28]. The induced protection against murine cysticercosis using the truncated KETc1 is encouraging, and allows us to propose the use of a new shortened peptide (9 amino acids) as part of a multipurpose vaccine. Regarding GK1, truncation of the last ten amino acids abolished its protective capacity. Nonetheless, since this peptide is almost identical between the analyzed cestode species, the 18-aa original version could be employed in a multipurpose polyvalent vaccine against the different cestodiases.

The KETc1 sequence merits additional comments. The clone that included KETc1 was identified by immunoscreening of a cDNA library constructed in UNi-ZAP XR vector, and contained an insert of 1.2 Kb. A rabbit antibody against a previously identified protective antigen fraction [28] was initially used to screen the library. KETc1 was then selected after being recognized by pooled sera from cysticercotic pigs during a second screening [28]. Unexpectedly, sequence analysis considering the one in frame with the lacZ gene and the adaptor sequence including the EcoRI restriction cloning site (GAATTCGGCACGAG) used to clone the 5′3′ cDNA fragments in the λZAP vector revealed a stop codon (TAA in *T. solium* and *E. multilocularis* and TGA in the others cestodes) 12 amino acids downstream of the reading frame. Despite this unexpected finding, the corresponding 11 amino-acid peptide synthetically produced plus an alanine at the 5′ end and a glycine at the 3′ end, demonstrated highly immunogenic properties and induced high levels of protection when used as immunogen against murine cysticercososis. This finding points to the correct interpretation of these data [16], [39].

Immunolocalization of the KETc1 and GK1 peptides provided valuable information. The two peptides were localized in the tegument of the adult and the larval stages of the different cestodes (Fig. 3 to [Fig pone-0011287-g004] 5). This localization could be critically related to their protective properties since the tegument is a metabolically active layer responsible for nutrient absorption and digestion. It also participates in defending the worm against the host's digestive enzymes. Furthermore, considering that the tegument's function and morphology is conserved in cestodes, it seems conceivable that KETc1 and GK1 expression across species is quite uniform [40], [41]. Moreover, it is also probable that both peptides could be detected in other organisms of medical relevance that belong to this monophyletic cestode taxon i.e. *Hymenolepis, Dyphillobotrium, Spirometra* [41], [42]. In addition, KETc1 in *T. crassiceps* ORF is detected in the outer surface of the tegument of the larval stage, indicating its presence in the microtriches of the tegument. These are ciliated sensory structures that increase the absorptive area of the tapeworm [40], [41], [43].

The additional localization in the parenchyma could also be related to critical parasite functions involved in the transport of nutrients from the tegument to the inner organs. The expression of these two epitopes in *T. solium* oncospheres should be noted since it is a parasite stage highly susceptible to immune attack [44]. It is highly feasible that these epitopes are also expressed in the oncospheres of the other cestodes, considering their functional and morphological similarities.

Importantly, besides the capacity to elicit specific immunity [19], [45], S3Pvac also elicits innate immunity by activating antigen-presenting cells [46], [47]. This finding reinforces the interest of including these peptides in a polyvalent vaccine.

Altogether, these results support the possible efficacy of KETc1 and GK1 if included in the contents of a multivalent vaccine against these cestodiasis. Further field trials using these two peptides against different cestodiasis will support their effectiveness in vaccination.

## Materials and Methods

### Ethics statement

Animal management for serum and parasite collection was performed according to the principles set forth in the Guide for the Care and Use of Laboratory Animals, Institute of Laboratory Animal Resources, National Council, Washington, D.C. 1996. This study was approved by the Ethics Committee of the Instituto de Investigaciones Biomédicas, Universidad Nacional Autónoma de México (UNAM).

### Parasites

The ORF strain of *T. crassiceps* has been maintained by serial intraperitoneal passages in BALB/cAnN female mice for more than 20 years at our Institute. Parasites for infections were harvested from the peritoneal cavity of mice, 1–3 months after inoculation of 10 cysticerci per mouse [37].

*T. crassiceps* WFU strain was isolated from a field mouse (*Peromyscus sp*.) captured in the state of Michigan, USA in August 1999 by Dr. Raymond Kuhn and maintained by intraperitoneal passage in female BALB/cAnN mice thereafter [48]. In December 1999 this strain was kindly donated by Dr. Kuhn to Dr. Kaethe Willms of the Medicine Faculty at the Universidad Nacional Autónoma de México, and thereafter to our research group in 2007. Since then, it has been maintained by intraperitoneal passage each 4–6 months by inoculation of 10 cysticerci using a 21G needle gauge. Cysticerci were recovered by cutting open the abdominal wall, extracting them and washing them with sterile saline solution. *T. crassiceps* WFU tapeworms were recovered by dissection of the intestine of outbred golden hamsters (*Mesocricetus auratus*) 20 days after oral infection with five cysts each.

*T. solium* cysticerci were dissected from skeletal muscle of highly infected pork carcasses 2 to 4 h after slaughter in an abattoir in Zacatepec, Morelos, Mexico. Segments from *T. solium* tapeworm and eggs were obtained from the feces of an infected carrier as previously described [16], [17].

*Taenia saginata* cysticerci were recovered from slaughterhouses in Guararapes, SP, Brazil from naturally infected cows identified during sanitary inspection. The adult tapeworm was recovered from a human carrier in Guerrero, Mexico, after specific medical treatment.

*E. granulosus* specimens were obtained from the liver of naturally infected pigs from slaughterhouses in the State of Morelos, Mexico. Parasites were extensively washed with sterile saline solution, dissected, and immediately stored in ethanol.

Metacestodes from *E. multilocularis* belonging to isolate H95 were recovered from experimentally infected Mongolian jirds (*Meriones unguiculatus*).

### Extraction of Genomic DNA (gDNA)

Metacestode genomic DNA from the ORF and WFU strains of *T. crassiceps*, *T. solium*, *E. granulosus*, and *T. saginata* which were excised from infected mice, pigs and cattle respectively, and purified using GenomicPrep kit (GE, Buckinghamshire, UK) following the protocol for DNA isolation of animal tissue. *E. multilocularis* DNA was isolated following a method reported elsewhere [49].

### PCR amplification and DNA sequencing

All PCR reactions were performed in a final volume of 50 µl, containing 5 µl of 10× PCR buffer (Invitrogen, Carlsbad, CA, USA) and 1 µl 100 mM deoxynucleoside triphosphates (Invitrogen), 1.7 µl of magnesium chloride, 1 µl of the corresponding forward and reverse primers at a concentration of 20 ng/µl and 0.5 µl of Taq DNA polymerase (Invitrogen). Five hundred nanograms of genomic DNA were used for each reaction. A positive control was employed consisting of the original sequences that codified for the respective peptides cloned in the plasmids pSK or pUI 235-5.1.

Primers employed for amplification of the homologous sequences of KETc1, and GK-1 (which is inserted into the KETc7 sequence) (Genbank Accession Number U31524) are shown in Table 3. A Palm Cycler (Corbett Life Science, Sydney, Australia) was used for amplification, and it included a denaturation cycle at 94°C for 3 min; 35 cycles at 94°C for 15 s, 30 s at the optimum annealing temperature for each peptide, and 72°C for 30 s. A final extension cycle at 72°C for 7 min was included. Optimum annealing temperatures were for KETc1 54°C and GK1 52°C. Amplification products were analyzed on a 2% agarose gel and visualized in UV light by ethidium bromide staining. DNA bands corresponding to the expected molecular weights were excised and purified using the QIAEX II kit (Qiagen).

**Table 3:** Primers used for PCR amplification of the KETc1 and GK-1 (KETc7) homologous sequences in *T. crassiceps* ORF, *T. crassiceps* WFU, *T. solium*, *T. saginata*, *E. multilocularis*, and *E. granulosus.*

Peptide	Primers	Sequences (5′-3′)
KETc1	KETc1 2294	GCTCCAATGTCTACACCAT
	K1b-AS	Universidad Nacional Autónoma de México, Distrito Federal, México,
GK-1	Sense primer	GCATTTATGCAGCCGCATCCTTCC
	A05	CTAAAGATTCTTCTTATCTTCTGGTTCCAT

Sequencing was done either in an ABI PRISM 310 Genetic Analyzer PE at the Instituto de Investigaciones Biomédicas, UNAM or in a Perkin Elmer/Applied Biosciences 3730 at the Instituto de Biotecnología, also at UNAM.

### Sequence analysis

Amino acid sequence alignments were performed using ClustalW/X 2.0 [50], T-Coffee [51], and BLAST[52]. cDNA sequence alignment was done using ClustalX and corrected by naked eye to make them consistent with those of amino acids using Seaview and eBiox/eBiotools.

dN/dS ratio determination was performed using codeml and YN00 from the PAML (v.4.2) package by Ziheng Yang [53], [54].

KETc1 cDNA and cDNA-derived amino acid alignments were divided in two parts to minimize the noise produced by sequence ambiguities (i.e, not all sequences are of the same size, (see Figure 1). For the sequence corresponding to GK1, only the bases that encode this peptide plus three previous amino acids were analyzed (Figure 2).

Genomic and cDNA sequences were translated to amino acids using Expasy's Translate Tool; the corresponding reading frame was determined based on the presence of the reported sequences for KETc1 and KETc7. Resulting sequences were compared to reported ones from *T. solium* (UNAM) and *E. multilocularis* (Sanger Institute) genomes using BLAST [52].

Accession numbers of the sequences are: for KETc7 in *T. crassiceps ORF* (Genbank Accession Number U31524), in *T. solium* (UNAM) (Genbank accession number EL745878), in *T. saginata* (Genbank Accession Number HM010764) and in *T. crassiceps* WFU (Genbank Accession Number HM010765). For KETc7 in *E. multilocularis* the homologues sequences were identified from the database of *Echinococcus* project (www.sanger.ac.uk/Projects/Echinococcus/), (Sanger Institute), contig 0011155 (subsequence 472–1560) and for *E. granulosus* (Genbank Accession Number HM010766),

For KETc1 in *Taenia crassiceps* ORF (Genbank Accession Number HM010769), in *T. solium* (UNAM) (Genbank accession number EL740346), in *T. saginata* (Genbank Accession Number HM010767) and in *T. crassiceps WFU* (Genbank Accession Number HM010768). Data for *E. granulosus* and *E. multilocularis* were downloaded from the database of *Echinococcus* project (www.sanger.ac.uk/Projects/Echinococcus/), (23187935 and contig 0006325 (subsequence 49202–49933), respectively).

### Peptide 3D-structure prediction

The 3D-structures of the relevant regions containing the conserved protection-inducing motifs were modeled using the HMMSTR Ab-initio prediction server [55], [56].

Results were obtained as .pdb files and further analyzed using Swis-PdbViewer and eBiox/eBiotools for MacOs X. Table 1 shows the sequences used to perform this analysis.

### Antibodies

Specific rabbit anti-KETc1 and anti-GK1 antisera were prepared by immunization of one white female New Zealand rabbit for each. Rabbits were bled before subcutaneous immunization with 100 µg of each synthetic peptide in Freund's incomplete adjuvant (Sigma), 5 times each 15 days. Ten days after the last immunization rabbits were bled and their sera were collected. The presence of specific antibodies was tested by ELISA using the respective synthetic peptides as antigens. The presence of specific antibodies was monitored by ELISA during the immunization schedule using the synthetic peptides as source of antigen according to a previously reported procedure, with minor modifications [17]. Polystyrene plates were irradiated with UV light prior to coating the target synthetic peptide to improve peptide attachment to the solid phase [57].

### Immunolocalization of the S3Pvac peptides

The distribution of S3Pvac peptides in the different cestodes was analyzed considering that it could provide information concerning their potential physiological functions in these parasites. The different parasite specimens were fixed and stained mainly following the procedure described by Montero et al., 2007 [58]. Specific rabbit antibodies were prepared as described above and employed as primary antibody. Non-specifically bound host proteins were dissociated from the different cestodes using a previously described procedure [27]. Briefly: vesicular fluid was obtained from cysts, washed thoroughly with ice cold PBS, and incubated with 50 mM glycine–HCl, pH 2.5; 0.1% Triton X-100; 0.15mMNaCl for 30 s. pH was restored by adding Tris–HCl, pH 9, followed by incubation in Zamboni solution for 72 hours. Afterwards the specimens were embedded in paraffin and six micrometer sections were cut. Slides were placed on poly-l-lysine (Sigma)-treated microslides. Peroxidase activity was blocked by treatment with 3% H_2_O_2_ in PBS for 10 min at room temperature. After washing with PBS, the slides were blocked with 5% BSA in PBS plus 0.1% Triton X-100 (pH 7.4) for 1 h at 37°C. Solutions were removed and the slides were incubated overnight at 4°C with sera from pre-immune rabbits and *T. crassiceps* infected mice, or specific rabbit antibodies against GK1 and KETc1 prepared as described above. These sera were employed as the primary antibody at a dilution of 1:1000 in 1% BSA in PBS plus 0.1% Triton X-100 (PBS/A-T). After washing three times in PBS/A-T, 5 min each, the slides were covered with a biotinylated goat anti-mouse IgG (ImmunO Universal Kit, MP Biomedicals, Eschwege, Germany) for 30 min at 37°C, rinsed with PBS/A-T, and treated with streptavidin-peroxidase conjugate (HRP conjugated, Universal Kit, MP Biomedicals, Eschwege, Germany) for 30 min at 37°C. Peroxidase activity was visualized by incubating the samples with 3′3-diaminobenzidine (DAB-Plus Kit, Zymed, South San Francisco, CA.). The slides were counterstained with Mayer's hematoxylin, dehydrated, cleared, mounted, and observed with an optical microscope (Nikon) using the MetaMorph Imaging System 4.5 software.

### Mice immunization and challenge

In order to determine the relevance of the few differences found between peptides regarding their protective capabilities, full sequences of GK1 and KETc1 and their respective truncated versions were tested for their protective capacity using a murine model of cysticercosis, which has been extensively employed to identify antigens of interest for vaccination. Eight female mice per experimental group, bred and kept in our animal facilities, were used for vaccine trials. The experiments reported herein were conducted according to the principles set forth in the Guide for the Use of Laboratory Animals, Institute of Laboratory Animal Resources, National Research Council, Washington, DC, 1996. The experimental protocols were approved by the Animal Care Committee of the Universidad Nacional Autónoma de México and the Guide for Care and Use of Experimental Animals was followed.

BALB/cAnN mice were subcutaneously immunized with 10 µg/mouse of KETc1, GK1, and their respective truncated peptides. Immunization was performed twice at 10-day intervals. Fifteen days after the second immunization, naïve and immunized mice were intraperitoneally challenged with 15 non-budding cysticerci of *T. crassiceps* (2 mm in diameter), in 0.9 ml of PBS. To determine the level of protection, mice were sacrificed thirty days after infection and the cysts inside the peritoneal cavity were harvested and counted under a stereoscopic microscope.

### Peptides

The *T. crassiceps* derived peptides KETc1 (APMSTPSATSVR) and GK1 (GYYYPSDPNTFYAPPYQ) were synthetically produced by AnaSpec Inc. A truncated peptide of KETc1: Ala Pro Met Ser Thr Pro Ser Ala Thr (APMSTPSAT) and another of GK1: Gly Tyr Tyr Tyr Pro Ser Asp Pro Asn Thr (GYYYPSDPNT) were synthesized by AnaSpec Inc. Peptide purity was >95% as judged by HPLC.

### Statistical Analysis

Statistical comparison of individual parasite intensities between groups was performed by Student's *t*-test. All statistical analyses were performed by the Instat software program (GraphPad, San Diego,Calif.). *P*<0.05 was considered statistically significant.
